# Mechanism Insights in Freeze–Thaw Process Impacting Cold Denaturation of Gluten Proteins During Frozen Storage

**DOI:** 10.3390/foods14173103

**Published:** 2025-09-05

**Authors:** Yang Li, Yilin Sun, Shuya Chen, Mingfei Li, Xiaowei Zhang, Yujie Lu

**Affiliations:** 1 School of Grain Science and Technology, Jiangsu University of Science and Technology, Zhenjiang 212001, China; 2 Anhui Province Key Laboratory of Functional Agriculture and Functional Food, Anhui Science and Technology University, Chuzhou 239000, China; 3 School of Food Science and Technology, Jiangnan University, Wuxi 214122, China

**Keywords:** gluten proteins, cold denaturation, frozen storage, freeze–thaw cycles, molecular dynamics simulations

## Abstract

Cold denaturation of gluten proteins during prolonged frozen storage or repeated freeze–thaw cycles can severely affect the quality of frozen cereal products. While both processes have been studied individually, their combined effects and underlying mechanisms remain unclear. This study systematically evaluated the hydration properties and conformational changes in gluten proteins stored at −73 °C and −23 °C, with or without freeze–thaw cycling. Compared to continuous storage, freeze–thaw cycles reduced water-holding capacity by 9.1–12.2% and increased oil-holding capacity by 5.3–10.3%, indicating aggravated structural damage. Ultra-low temperature storage (−73 °C) suppressed ice crystal growth, preserved hydration, and limited hydrophobic residue exposure. Spectroscopic analyses revealed a temperature-dependent shift from α-helices to β-sheets and β-turns, which was accelerated by freeze–thaw cycles. Enhanced hydrophobic interactions and tryptophan exposure further indicated destabilization. Molecular dynamics simulations showed that increased hydrogen bonding between proteins and water contributed to unfolding at low temperatures, while temperature fluctuations intensified denaturation through repeated hydrogen bond breakage and reformation. These results underscore the critical role of thermal instability in cold denaturation and offer mechanistic insights for improving cryoprotection strategies in frozen food systems.

## 1. Introduction

Freezing causes a shift in the conformation of natural proteins, which weakens their stability and functionality. This is called the cold denaturation of proteins [1]. With the progress in plant-based meat, gluten proteins are increasingly acknowledged as an essential source [2,3,4,5]. Additionally, glutenin could function as a scaffold for cultured meat [6]. Nevertheless, frozen storage and freeze–thaw cycle processes will disturb the structure of gluten proteins and affect the quality of plant-based meat. These are primarily due to the alteration of molecular conformations of gluten proteins. The depolymerization of gluten fractions happens by reducing disulfide (S-S) bridges to thio (SH) groups and S-S/SH interexchange reactions, and noncovalent interactions such as hydrogen bonding and hydrophobic interaction further drive the conversions of secondary structures during freezing [7].

Freeze–thaw cycles, when compared to frozen storage, mimic the most extreme scenario of quality degradation, causing a more pronounced depolymerization of glutenin macropolymer. Moreover, Liang et al. observed that the molecular structure of the gluten proteins underwent significant variations at different freezing temperatures. Gluten structure can degrade due to water migration and ice crystal formation, particularly under suboptimal freezing conditions such as fluctuating or moderately low temperatures (e.g., −23 °C). In contrast, ultra-low temperatures (e.g., −73 °C) are generally protective, although certain stressors, such as repeated freeze–thaw cycles, may still compromise structural integrity [8]. Dai et al. observed that the water-holding capacity of gluten proteins was higher at low temperatures with the same number of freeze–thaw cycles. However, at the same temperature, the water-holding capacity, non-freezable water content, and viscoelasticity gradually decreased with an increase in the number of freeze–thaw cycles [9]. These findings suggest that the freezing temperature is a key factor in gluten structure and properties, whether in frozen storage or during freeze–thaw cycles. Smaller ice crystal sizes were observed at ultra-low temperatures due to the rapid rate of freezing, resulting in decreased damage to frozen food [10]. Moreover, current research primarily examines the impact of frozen storage or freeze–thaw cycles on gluten proteins individually, and the impact differences in freeze–thaw cycles and frozen storage on molecular conformations of gluten proteins are relatively lacking. The mechanism by which temperature fluctuations exacerbate the degradation of frozen proteins requires further clarification.

This study provides an integrated investigation of the cold denaturation of gluten proteins under the combined effects of long-term frozen storage and freeze–thaw cycles superimposed on frozen storage across different storage temperatures. Through both experimental analysis and molecular dynamics simulations, some novel perspectives are presented. This study provides new insights into the synergistic effects of these two stressors. The water-holding and oil-holding capacities of gluten proteins, freezable water content, and surface hydrophobicity were comparatively tracked during frozen storage and freeze–thaw cycles at different temperatures. Furthermore, the secondary structure and amino acid microenvironment were analyzed. Finally, the molecular conformations of gluten proteins at varying temperatures were analyzed using molecular dynamics simulations to elucidate the conformational changes triggered by temperature fluctuations. This investigation aimed to provide insights into the mechanisms underlying cold denaturation during frozen storage and denaturation exacerbated by freeze–thaw cycles. The integration of experimental analysis and simulation techniques will establish a more comprehensive theoretical basis for comprehending the cold denaturation of gluten proteins and provide novel insights for quality improvements in frozen dough and plant-based meat.

## 2. Materials and Methods

### 2.1. Materials

Commercial flour was purchased from New Land Group (Xinxiang, China). All other chemicals were obtained from Sinopharm Chemical Reagent Co. (Shanghai, China).

### 2.2. Preparation of Gluten Samples

The gluten proteins were extracted according to our previous method [11]. In this study, the temperature of −23 °C (250 K) was selected as it reflects a typical commercial freezing condition used in the food industry, balancing energy efficiency and product preservation. Subsequently, we applied −73 °C (200 K), a temperature known to have minimal effects on protein structure and frequently employed in biomedicine [12]. In frozen-storage groups, 5 g of wet gluten samples were weighed and then placed in environments set at −73 °C (200 K) and −23 °C (250 K) for 14, 28, 42, and 56 days, respectively. In the groups of freeze–thaw cycles superimposed on frozen storage, the 5 g wet gluten samples were frozen at −73 °C (200 K) and −23 °C (250 K) to the 14th, 28th, 42nd, 56th days followed by thawing at 25 °C for 4 h, then continued to freeze to the same days with the frozen-storage groups [13]. After specific periods of frozen storage and each freeze–thaw cycle, the gluten samples were freeze-dried and screened through 80 mesh for further analysis.

### 2.3. Water-Holding and Oil-Holding Capacity Analysis

Protein powder (0.1 g, *M*_1_) was weighed and transferred into a centrifuge tube. Subsequently, 2 mL of water was incrementally added, and the mixture was stirred using a glass rod for 30 min until complete dissolution. The solution was then centrifuged at 10,000× *g* for 5 min. The supernatant was removed, and the weight of the precipitate (*M*_2_) was determined. The water-holding capacity (WHC) of gluten proteins was calculated as follows [14].

$$\mathrm{WHC}=\frac{M_{2}-M_{1}}{M_{1}}$$

Protein (0.25 g, *M*_1_) was placed in a centrifuge tube and weighed. Subsequently, 2.5 mL of rap oil was added to the centrifuge tube and stirred rapidly for thorough mixing. After centrifugation at 10,000 rpm for 5 min, the supernatant was discarded, and the weight of the precipitate (*M*_2_) was determined. The oil-holding capacity (OHC) was calculated as follows [15].

$$\mathrm{OHC}=\frac{M_{2}-M_{1}}{M_{1}}$$

### 2.4. Freezable Water Content Analysis

The freezable water content of gluten samples was determined using a Differential Scanning Calorimetry (DSC) instrument (DSC25, TA Discovery, New Castle, DE, USA). Preceding the testing phase, gluten samples ranging from 10 to 20 mg were isolated from the thawed gluten proteins and then sealed tightly within aluminum DSC pans. The pans were pre-chilled in a refrigerator at −20 °C for 12 h for pre-equilibration [16]. Subsequently, the samples were expeditiously transported to the DSC instrument. Equilibration of samples at −20 °C for 5 min was followed by heating to 10 °C at a rate of 1 °C per minute. By means of the TA Rheology System Software Muse, the melting enthalpy (Δ*H*, in J/g of water) was computed [17].

### 2.5. Surface Hydrophobicity Analysis

After being dissolved in a 0.5 mol/L acetic acid solution at 20 °C for 2 h, the freeze-dried gluten samples underwent centrifugation at 10,000× *g* for 15 min. The supernatant was then diluted to a concentration of 0.01 to 1 mg/mL using the aforementioned acetic acid solution. Following this, 50 µL of ANS solution (8 mmol/L, 0.1 mol/L phosphate buffer, pH 5.8) was uniformly combined with 8 mL of the gluten protein solution [18]. After each mixture was kept in a dark environment for 20 min, the fluorescence intensity was determined through a fluorescence chromatograph (F-7000, Hitachi, Tokyo, Japan). The excitation wavelength, the emission wavelength, and the slit were set as 390 nm, 470 nm, and 5 nm, respectively. Plotting the curve of fluorescence intensity in relation to protein concentration enabled the determination of the surface hydrophobicity index of gluten samples, which could be derived from the slope of the initial stage of the curve.

### 2.6. Fourier Transform Infrared (FT-IR) Spectroscopy

A 1:50 ratio of freeze-dried gluten sample and potassium bromide was blended together and then pulverized into a fine powder using an agate mortar. Afterward, the powder was condensed into a transparent sheet and examined with an FT-IR spectrometer (Nicolet IS10, Thermo Fisher Scientific Inc., Madison, WI, USA). The scanning time and resolution were set to 32 and 4 cm^−1^, respectively. The wave number ranged from 400 to 4000 cm^−1^. The collected data were subsequently analyzed and processed using OMNIC (V.8.2, Thermo Fischer Scientific Inc., Madison, WI, USA) and Peak Fit software (V.4.12, Systat Software Inc., San Jose, CA, USA). In detail, the spectrum of Amide Ⅰ band (1590~1710 cm^−1^) was selected, followed by baseline correction, Gaussian smoothing, and deconvolution, and the absorption peak of the responding secondary structure was quantified [19].

### 2.7. Raman Spectroscopy

By means of Raman spectroscopy (LabRAM HR Evolution, HORIBA Scientific Inc., Palaiseau, France), the aromatic amino acid environment was examined, featuring the tyrosine doublet (I_854_/I_830_) and the tryptophan band (I_755_). A small amount of gluten sample was evenly applied to a glass slide wrapped in tin foil. A clear image was found under the microscope, then spectra were collected over a range of 200~4000 cm^−1^ with an excitation wavelength of 532 nm. At least 6 points were selected for each sample. The spectral baseline correction was performed using LabSpec 6 software (HORIBA Scientific Inc., Palaiseau, France).

### 2.8. Fluorescence Quenching Experiments

Following extraction with a 1 mL solution of 50 mmol/L of acetic acid at room temperature for 1 h, the freeze-dried gluten sample underwent centrifugation at 10,000× *g* and 25 °C for 15 min. Dilution of the supernatant with the extraction solvent was carried out to achieve a concentration of 1 mg/mL. The fluorescence of gluten proteins was quenched using an 8 mol/L acrylamide solution prepared with Tris-HCl (10 mmol/L, pH 7.7). The solvent accessibility and polarity of the Trp residue microenvironment were assessed through quenching experiments utilizing acrylamide as the quencher [20]. A 2 mL solution of gluten proteins was prepared in 10 mL volumetric flasks. Varying amounts of acrylamide were added to the solution, resulting in acrylamide concentrations ranging from 0 to 5 μmol/L. The solutions were incubated for 5 min to achieve equilibrium. The excitation wavelength was fixed at 292 nm, and the emission spectra were recorded in the range of 310 to 410 nm. The fluorescence quenching data were analyzed using the Stern–Volmer equation [7].

### 2.9. Molecular Dynamics Simulations

Due to the high polymerization and complexity of gluten proteins, the crystal structure analysis was not realized. In our prior study, we observed that α-helices in gluten proteins were susceptible to freezing treatments, and the alterations in their conformation were closely linked to protein quality [11,21]. Furthermore, Tang et al. conducted molecular docking studies revealing a tendency for cryoprotective polysaccharide monomers to bind with α-helix structures in simulated gluten proteins [14,15], also reflecting the importance of α-helices. Consequently, our study selected key α-helix structures from *Triticum aestivum* wheat to create PDB files for further analysis. Molecular dynamics simulations were performed using the GROMACS software suite (v.5.1.4). The simulation systems were established, and the results analysis was performed based on our prior research [11,21].

### 2.10. Statistical Analysis

All measurements were conducted in triplicate and the mean ± standard deviation (SD) was reported. Duncan’s multiple range test was conducted using SPSS software (version 26, SPSS Inc., Chicago, IL, USA). Statistical significance was determined if the probability value was *p* < 0.05.

## 3. Results

### 3.1. Changes in Water-Holding and Oil-Holding Capacities

**Figure 1:**
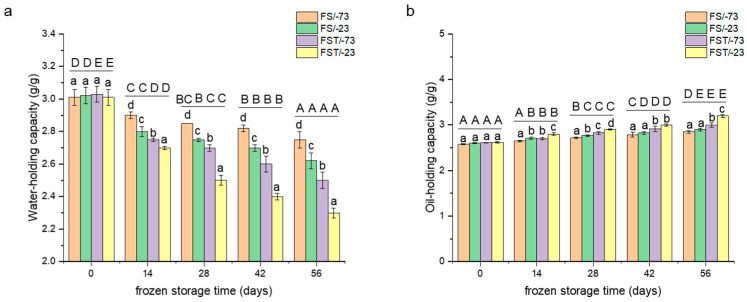
Changes in water-holding capacity (**a**) and oil-holding capacity (**b**) of gluten proteins. FS/-73, gluten proteins stored at −73 °C; FS/-23, gluten proteins stored at −23 °C; FST/-73, gluten proteins after freeze–thaw cycles during frozen storage at −73 °C; FST/-23, gluten proteins after freeze–thaw cycles during frozen storage at −23 °C. Mean values with different lowercase letters are significantly different (*p* < 0.05) with different treatments, and different uppercase letters are significantly different (*p* < 0.05) in frozen storage time. Error bars represent mean standard deviations of triplicate determinations.

The water-holding capacity (WHC) of gluten proteins is a key functional attribute that indirectly reflects the uniformity and stability of their internal network structure. As shown in Figure 1a, WHC progressively declined with increasing frozen-storage duration. However, gluten samples stored at −73 °C exhibited significantly higher WHC values than those stored at −23 °C (*p* < 0.05), suggesting that ultra-low temperatures may suppress ice crystal growth and thereby reduce structural damage to gluten proteins [22]. The results were in accordance with the theory of ice nucleation and growth [23]. In addition, freeze–thaw treatments induced more pronounced structural disruption, leading to a steeper decline in WHC. Specifically, after four freeze–thaw cycles, WHC values were 9.1% and 12.2% lower than those of samples stored continuously for 56 days at −73 °C and −23 °C, respectively. This deterioration is likely attributable to the thawing process, which promoted the transformation of loosely bound water into free water. Upon refreezing, this free water formed additional ice crystals that exacerbated microstructural damage to the gluten network [24].

The oil-holding capacity (OHC) of gluten proteins exhibited a negative correlation with the WHC [14,15]. As shown in Figure 1b, OHC gradually increased with extended frozen storage, in contrast to the decreasing trend observed for WHC. Higher storage temperatures (−23 °C) significantly elevated OHC values (*p* < 0.05), likely due to more pronounced structural alterations in the protein matrix, such as depolymerization and increased exposure of hydrophobic amino acid residues, which enhanced oil-binding capacity [11]. Notably, freeze–thaw cycles further intensified the increase in OHC over the same storage duration (*p* < 0.05). After four freeze–thaw cycles, the OHC values were 5.3% and 10.3% higher than those of samples stored continuously at −73 °C and −23 °C for 56 days, respectively. This highlighted the substantial impact of temperature fluctuations on protein functionality [9]. Furthermore, gluten proteins stored at −23 °C consistently exhibited higher OHC than those stored at −73 °C (*p* < 0.05), reinforcing the notion that elevated storage temperatures promoted the exposure of hydrophobic regions. Collectively, these results suggested that freeze–thaw cycling and higher storage temperatures both enhanced hydrophobicity in gluten proteins, as reflected by increased OHC [24].

### 3.2. Changes in Freezable Water Content

**Figure 2:**
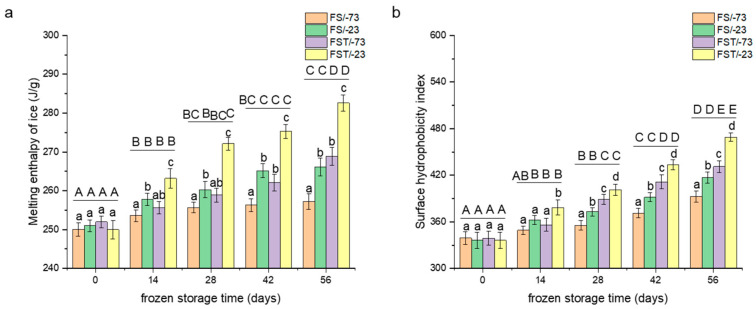
Changes in surface hydrophobicity (**a**) and freezable water content (**b**) of gluten proteins. FS/-73, gluten proteins stored at −73 °C; FS/-23, gluten proteins stored at −23 °C; FST/-73, gluten proteins after freeze–thaw cycles during frozen storage at −73 °C; FST/-23, gluten proteins after freeze–thaw cycles during frozen storage at −23 °C. Mean values with different lowercase letters are significantly different (*p* < 0.05) with different treatments, and different uppercase letters are significantly different (*p* < 0.05) in frozen storage time. Error bars represent mean standard deviations of triplicate determinations.

In DSC analysis, Δ*H* corresponds to the amount of energy absorbed during the phase transition of ice to liquid water. Since only the freezable portion of water contributes to this thermal event, Δ*H* is directly proportional to the freezable water content in the sample. A reduction in Δ*H* indicates a lower amount of freezable water, suggesting stronger water–protein interactions or increased water immobilization [14]. As shown in Figure 2a, melting enthalpy increased with longer frozen-storage duration, indicating a progressive rise in freezable water content. At the same time point, samples stored at −23 °C exhibited significantly higher Δ*H* values than those stored at −73 °C, likely due to the formation of less stable ice crystal structures at higher temperatures, which permitted greater mobility of water molecules [10]. Moreover, under identical storage temperatures, samples subjected to freeze–thaw cycles showed significantly higher Δ*H* than those under continuous frozen storage (*p* < 0.05), particularly after 42 days. Specifically, after freeze–thaw treatment, Δ*H* values were 4.5% and 6.2% higher than those recorded after 56-day continuous storage at −73 °C and −23 °C, respectively. This suggests that freeze–thaw cycling increased the amount of freezable water in gluten proteins, thereby intensifying ice crystal formation and aggravating structural damage [14]. In contrast, prolonged continuous storage at −73 °C did not lead to significant changes in freezable water content (*p* > 0.05), supporting the notion that ultra-low temperatures better preserved protein integrity by limiting water migration [10]. The superior cryoprotective effect of ultra-low temperatures offered practical insights for preserving protein structure in frozen food systems. Additionally, changes in freezable water content were closely associated with water-holding capacity. A reduction in WHC facilitated the release of previously bound water from gluten proteins, which then became part of the freezable water fraction. Upon freezing, this free water contributed to the formation of additional ice crystals, further destabilizing the protein matrix [25].

### 3.3. Changes in Surface Hydrophobicity

In order to further quantify the hydrophobicity variations in gluten proteins during frozen storage, the surface hydrophobicity index was tracked using the fluorescent probe ANS-binding method. The surface hydrophobicity indexes of gluten proteins at −73 °C were significantly lower than those at −23 °C (*p* < 0.05), as illustrated in Figure 2b. Upon extending continuous frozen storage to 56 days, the surface hydrophobicity index of gluten proteins exhibited increments of 15.7% and 24.0% compared with native proteins, respectively, at −73 °C and −23 °C. With the same frozen-storage period, the surface hydrophobicity of gluten proteins through freeze–thaw cycles showed increments of 27.2% and 39.5%, indicating that such cycles resulted in more substantial harm to gluten proteins and more noticeable alterations in surface hydrophobicity (*p* < 0.05).

The above analysis indicates that freeze–thaw cycles significantly exacerbated the deterioration of the hydration properties of gluten proteins. Unlike continuous frozen storage, the dynamic mechanical stresses arising from repeated ice formation and melting impose greater structural strain. Our previous study demonstrated a strong correlation between hydration properties and conformational behaviors [11,21], underscoring the need to further explore the impact of freeze–thaw cycles on conformations of gluten proteins during frozen storage.

### 3.4. Changes in Secondary Structure

**Figure 3:**
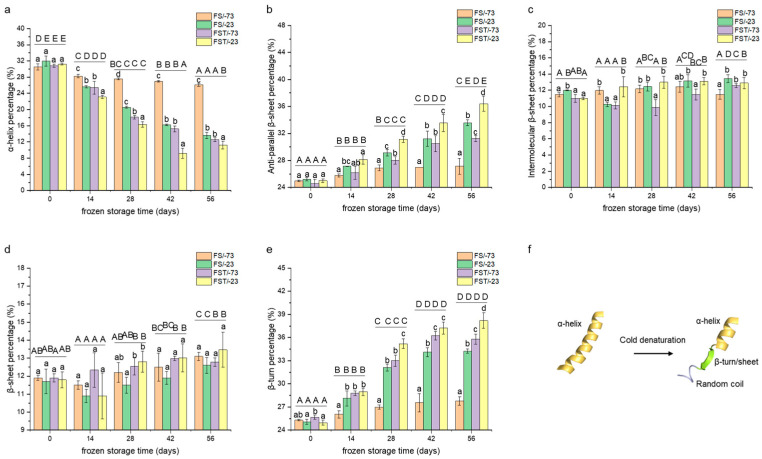
Changes in the secondary structure of gluten proteins. (**a**) α-helix percentage; (**b**) β-turn percentage; (**c**) anti-parallel β-sheet percentage; (**d**) β-sheet percentage; (**e**) intermolecular β-sheet percentage; (**f**) secondary structure transformations of gluten proteins through cold denaturation. FS/-73, gluten proteins stored at −73 °C; FS/-23, gluten proteins stored at −23 °C; FST/-73, gluten proteins after freeze–thaw cycles during frozen storage at −73 °C; FST/-23, gluten proteins after freeze–thaw cycles during frozen storage at −23 °C. Mean values with different lowercase letters are significantly different (*p* < 0.05) with different treatments, and different uppercase letters are significantly different (*p* < 0.05) in frozen storage time. Error bars represent mean standard deviations of triplicate determinations.

The alterations in the secondary structure of gluten proteins were assessed by an FT-IR spectrum. According to the corresponding assignments of secondary structure in the Amide I band (1600~1700 cm^−1^) [19], the percentages of the corresponding peak area to the total Amide I band area were calculated in Figure 3. As shown in Figure 3a, upon freezing and frozen storage, α-helices are most likely to undergo conformational transformations to maintain the structural stability of the overall gluten proteins [26]. During the initial 28 days of frozen storage, ordered α-helices were mainly converted into anti-parallel β-sheets and β-turns (Figure 3b,e). The observed conversion of α-helices to β-sheets and β-turns during freezing can be attributed to the destabilization of intramolecular hydrogen bonds that stabilize α-helical structures. Low temperatures and ice formation reduce the flexibility of the protein backbone and promote intermolecular hydrogen bonding and aggregation, conditions that favor β-sheet formation. Additionally, β-turns may form as a compensatory structure in response to local unfolding, contributing to altered compactness and hydration behavior. Frozen storage to the 56th day further elevated the proportion of intermolecular β-sheets and β-sheets at the expense of α-helices (Figure 3c,d). The increase in β-sheets led to a more hydrophobic and rigid structure [19], which was consistent with the alterations in its interactions with water. Additionally, α-helix percentages of gluten proteins at the same storage period and temperature were significantly decreased after freeze–thaw cycles (*p* < 0.05) (Figure 3a). Similar trends were observed for anti-parallel β-sheets and β-turns (Figure 3b,e). In contrast, the changes in intermolecular β-sheets and total β-sheets caused by freeze–thaw cycling were comparatively minor, suggesting that these β-sheet structures possessed greater resistance to freeze-induced denaturation [27].

Overall, frozen storage induced a reduction in α-helix percentage and the exposure of hydrophobic amino acid residues, with a decreased hydration capacity. Notably, freeze–thaw-cycles superimposed on frozen-storage conditions further intensified secondary structure disruption, even when samples were initially stored at −73 °C prior to thawing. These findings support the conclusion that extreme temperature fluctuations are a primary driver of conformational destabilization in gluten proteins during frozen processing [9].

### 3.5. Changes in Aromatic Amino Acid Microenvironment

**Figure 4:**
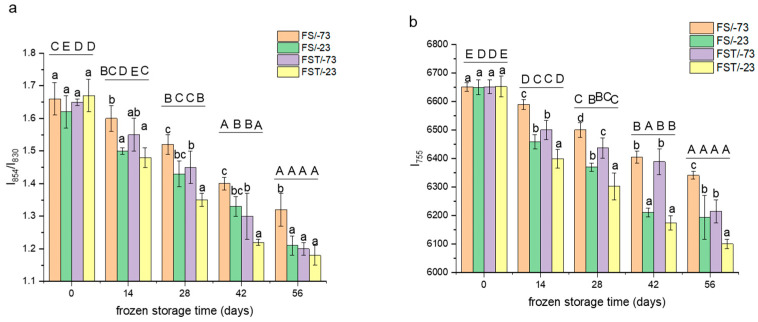
Changes in the microenvironment of the tryptophan and tyrosine of gluten proteins. (**a**) Raman absorbance at 755 cm^−1^ (tryptophan); (**b**) Raman absorbance ratio of 854 and 830 cm^−1^ (tyrosine pair). FS/-73, gluten proteins stored at −73 °C; FS/-23, gluten proteins stored at −23 °C; FST/-73, gluten proteins after freeze–thaw cycles during frozen storage at −73 °C; FST/-23, gluten proteins after freeze–thaw cycles during frozen storage at −23 °C. Mean values with different lowercase letters are significantly different (*p* < 0.05) with different treatments, and different uppercase letters are significantly different (*p* < 0.05) in frozen storage time. Error bars represent mean standard deviations of triplicate determinations.

To further verify the effects of temperature on the conformations of gluten proteins, the microenvironments of particular aromatic amino acids were analyzed, which could be reflected by the position and intensity of Raman absorption peaks [28]. The ratio of intensities (I_854_/I_830_) associated with the tyrosine peaks at 854 cm^−1^ and 830 cm^−1^ is indicative of the hydrogen bonding interactions involving the phenol hydroxyl group [7]. An I_854_/I_830_ value below 0.9 indicates that tyrosine residues are predominantly located in hydrophobic environments, whereas values above 0.9 suggest their exposure to polar environments [29]. As depicted in Figure 4a, the I_854_/I_830_ ratios for all gluten samples fell within the range of 1.1 to 1.8, suggesting the presence of weak hydrogen bonding interactions. With prolonged frozen storage, the I_854_/I_830_ ratios significantly decreased (*p* < 0.05), indicating a transition of the tyrosine microenvironment from polar to more non-polar conditions. After 56-day frozen storage, the I_854_/I_830_ ratios of gluten proteins after the freeze–thaw cycles were 9.1% and 2.5% lower than those undergoing continuous freezing at −73 °C and −23 °C, respectively. These results suggest that abrupt temperature fluctuations compromised the conformational stability of gluten proteins [30]. The relatively stable I_854_/I_830_ values observed during continuous storage at −73 °C might be attributed to the formation of fine, needle-like ice crystals [22], which exerted less mechanical stress on the protein matrix. As a result, gluten proteins stored continuously at −73 °C for 56 days retained significantly higher I_854_/I_830_ values than other treatment groups (*p* < 0.05). However, this structural preservation was notably diminished following freeze–thaw treatment, which increased the exposure of hydrophobic residues such as tyrosine. In contrast, the absolute I_854_/I_830_ ratios of gluten proteins stored at −23 °C remained relatively close across different treatments, resulting in smaller decreases.

The Raman absorbance at 755 cm^−1^ stemmed from the vibration of the anthracene ring in tryptophan, which could reflect microenvironment variations in tryptophan, whose decrements indicated that tryptophan was exposed from a non-polar environment [31]. As shown in Figure 4b, the I_755_ values of the 56-day frozen gluten proteins at −73 °C and −23 °C were 4.7% and 6.9% lower than those of non-treated proteins, respectively. Additionally, after 56-day frozen storage and freeze–thaw cycles at −73 °C and −23 °C, the values were 6.6% and 8.3% lower than those of non-treated proteins, respectively. The ultra-low frozen-storage temperature was more conducive to maintaining the conformations of gluten proteins, but the freeze–thaw treatment appeared to inflict damage on the internal interactions of gluten proteins, reducing the conformational stability during frozen storage [32].

### 3.6. Fluorescence Quenching Analysis 

**Figure 5:**
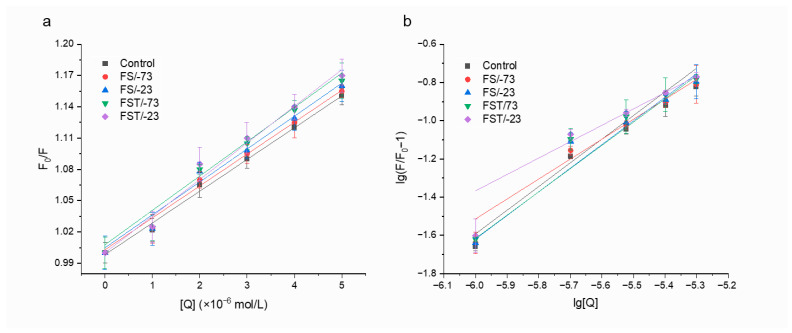
The Stern–Volmer curves (**a**) and the double-logarithm curves of lg (F_0_-F)/F vs. lg[Q] (**b**) for gluten proteins with acrylamide after frozen storage and freeze–thaw cycles. F_0_ and F are the fluorescence intensities in the absence and presence of the quencher, respectively; [Q] is the concentration of the quencher. Control, fresh gluten proteins; FS/-73, gluten proteins after 56-day frozen storage at −73 °C; FS/-23, gluten proteins after 56-day frozen storage at −23 °C; FST/-73, gluten proteins through freeze–thaw cycles during 56-day frozen storage at −73 °C; FST/-23, gluten proteins through freeze–thaw cycles during 56-day frozen storage at −23 °C.

Fluorescence quenching experiments were conducted with acrylamide as a quencher to investigate the conformational changes in gluten proteins induced by various freezing treatments [7]. Fluorescence quenching can be categorized into dynamic and static quenching, both of which were significantly influenced by temperature. The mechanism of dynamic quenching involves energy loss due to collisions between the quencher and the fluorophore, whereas in static quenching, a complex is formed between the quencher and fluorophore molecules in their ground state. Wang et al. reported that the quenching of acrylamide on gluten proteins was attributed to static quenching [7]. Thus, the Stern–Volmer equation was used to analyze the quenching process. As depicted in Figure 5, a strong linear relationship was observed between F0/F and [Q], with the Stern–Volmer quenching constants outlined in Table 1. The Ksv values of gluten proteins after 56-day frozen storage at −73 °C and −23 °C increased by 3.5% and 6.5%, respectively, compared with the Control protein. Significant changes (*p* < 0.05) occurred following freeze–thaw cycles, with Ksv values of gluten proteins increasing by 11.2% and 14.1% at −73 °C and −23 °C, respectively. It is probable that the disrupted gluten structure exposed the internal Trp and consequently increased the binding capacity of acrylamide [7]. Furthermore, the gluten proteins exhibited significantly increased K_b_ values (*p* < 0.05) following freeze–thaw cycles, indicating a heightened capacity to interact with acrylamide. An n value of approximately 1 suggested that acrylamide interacted with gluten proteins through a separate binding site [33]. This n value remained relatively unaffected by frozen storage and freeze–thaw cycle treatments. The fluorescence quenching analysis further confirmed the conformational transformations of gluten proteins during frozen storage and the aggravated effects of the freeze–thaw process. Next, the mechanism will be explored.

### 3.7. Molecular Dynamics Simulation Analysis

#### 3.7.1. Structural Stability and Flexibility

**Figure 6:**
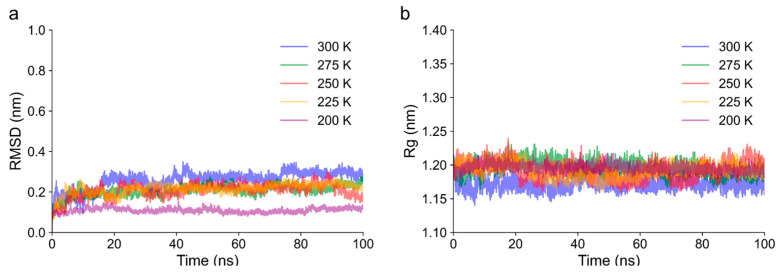
Trajectories of root-mean-square deviation (RMSD) (**a**) and radius of gyration (Rg) (**b**) of proteins at 300 K, 275 K, 250 K, 225 K, and 200 K.

To further investigate the differences in the cold denaturation of gluten proteins induced by frozen storage and freeze–thaw cycles, a glutelin structure obtained from AlphaFold Protein Structure Database was used to obtain the α-helix fragments sensitive to low temperature, followed by MD simulations at 300 K, 275 K, 250 K, 225 K, and 200 K. The root-mean-square deviation (RMSD) measures the average atomic displacement of the simulated structure from the initial structure. The radius of gyration (Rg) quantifies the conformational densification of a simulated protein, which could reflect the folding and unfolding process of the protein. Based on the RMSD trajectory in Figure 6a, equilibrium was reached by all simulation systems within 100 ns. The system at 200 K exhibited the lowest RMSD values among the tested temperatures (Figure 6a), while its Rg value increased significantly (Figure 6b). These findings suggest that lower temperatures reduced molecular mobility, resulting in smaller structural deviations but greater spatial expansion due to the strengthening of the hydrogen bonds of water–protein molecules and the weakening of hydrophobic interactions [34]. Essentially, the protein was locked into a partially unfolded or expanded state with limited dynamic rearrangement, resulting in a lower RMSD but higher Rg.

#### 3.7.2. Interaction with Water Molecules

**Figure 7:**
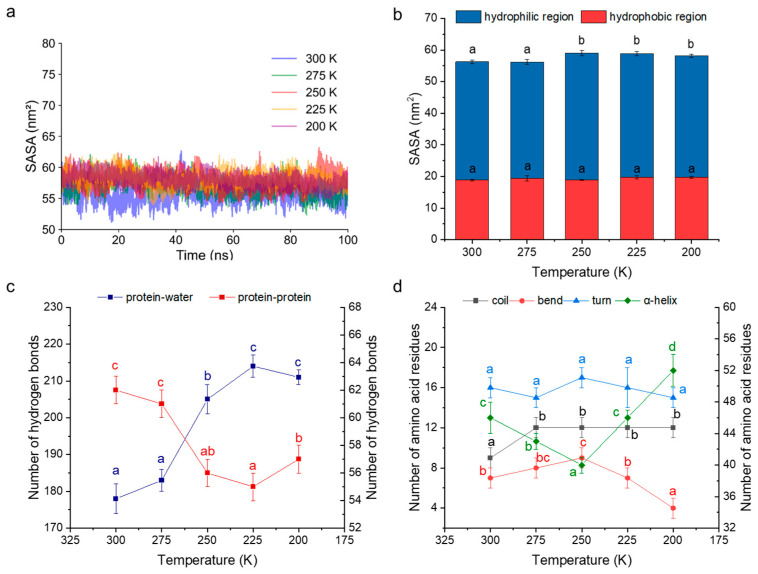
Trajectories of solvent-accessible surface area (SASA) (**a**), SASA analysis (**b**), changes in the number of hydrogen bonds (**c**), and changes in the number of amino acid residues (**d**) of protein at 300 K, 275 K, 250 K, 225 K, and 200 K. In (**c**), the left axis represents the number of hydrogen bonds between the protein and water; the right represents the number of hydrogen bonds between the protein molecules. In (**d**), the left axis shows the number of amino acid residues involved in coils, bends, and turns; the right shows the number of amino acid residues involved in the α-helix.

The solvent-accessible surface area (SASA) of a protein refers to the portion of its molecular surface exposed to the surrounding solvent during simulation. An increase in SASA generally indicates a greater exposure of amino acid residues and an enhanced potential for interactions with water molecules [35]. As shown in Figure 7a,b, all SASAs varied between 55 nm^2^ and 60 nm^2^ at different temperatures, with no significant difference in the hydrophobic region areas among the temperature groups (*p* > 0.05). Despite this, the hydrophilic areas at 250 K, 225 K, and 200 K showed a clear increase, resulting in a significant overall SASA increase (*p* < 0.05) in comparison to those at 300 K and 275 K. This increase was likely attributable to protein structural loosening at lower temperatures.

This observation aligns with the increased number of hydrogen bonds formed between protein and water molecules at 250 K, 225 K, and 200 K (Figure 7c). It is therefore speculated that the decreased kinetic energy of water molecules at low temperatures enhances protein–water hydrogen bonding while weakening intramolecular protein–protein hydrogen bonding, thereby promoting the unfolding of α-helical structures [1]. Previous studies have shown that ultra-low temperatures rapidly suppress water mobility, enhance hydrogen bonding among water molecules, and promote the formation of fine ice crystals [10]. Interestingly, in our simulation, the number of protein-protein hydrogen bonds increased at 200 K compared to 250 K, suggesting that extremely low temperatures may attenuate the influence of solvent molecules on secondary structures, thereby stabilizing protein conformations to a greater extent. This trend is consistent with results from secondary structure analysis. In addition, fluctuations in temperature ranging from 250 K to 300 K and returning to 250 K led to notable modifications in hydrogen bond formations and breaking, involving protein–protein and protein–water interactions. This provides molecular evidence for further damage of freeze–thaw cycles on gluten protein conformations at −23 °C during frozen storage.

Although the number of protein–water hydrogen bonds was significantly higher at 200 K than at 250 K (*p* < 0.05), this did not translate to more pronounced effects on gluten proteins in experimental observations. This discrepancy may stem from limitations of the simulation, which only considers solvent molecules in close proximity to the protein. In reality, rapid freezing at extremely low temperatures could mitigate the adverse effects of ice formation and recrystallization. Future studies should incorporate the phase transition behavior of water to better represent freezing dynamics in protein systems.

#### 3.7.3. Conformational Transformation Process

The number of amino acid residues involved in a protein secondary structure was analyzed, as shown in Figure 7d. As the temperature decreased from 300 K to 250 K, the number of residues forming α-helices reached a minimum. Interestingly, with a further temperature reduction, the α-helix content increased, with the value at 200 K being approximately 30% higher than at 250 K. This pattern closely aligned with FT-IR experimental results observed during frozen storage at −73 °C and −23 °C. In contrast, residues contributing to coil and bend structures exhibited inverse trends, while the distribution of residues in turn structures appeared more variable and less predictable.

**Figure 8:**
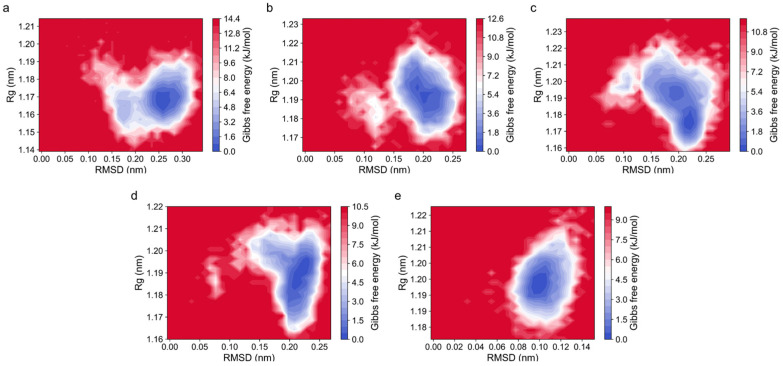
Gibbs free energy landscapes of proteins at 300 K (**a**), 275 K (**b**), 250 K (**c**), 225 K (**d**), and 200 K (**e**).

To gain deeper insight into the thermodynamic aspects of these conformational transformations, Gibbs free energy landscapes were constructed at various temperatures (Figure 8). At 300 K, the Rg fluctuation was in the range of 1.15~1.19 (Figure 8a). As the temperature decreased, the Rg in the low-energy (blue) region was prolonged. A new energy trap around Rg 1.19~1.20 emerged when approaching 273 K (Figure 8b), and this region broadened at 250 K (Figure 8c). These changes suggest that enthalpic contributions from hydrogen bond formation may promote the stabilization of specific conformational states at lower temperatures [36]. Interestingly, when the temperature continued decreasing to 225 K, there was one energy trap observed (Figure 8d), while the landscape at 200 K resembled that at 300 K (Figure 8e), indicating that ultra-low temperatures may help preserve native protein conformations. Collectively, these observations provide theoretical support for the application of rapid freezing and low-temperature storage as strategies to improve the structural preservation of gluten proteins.

## 4. Discussion

**Figure 9:**
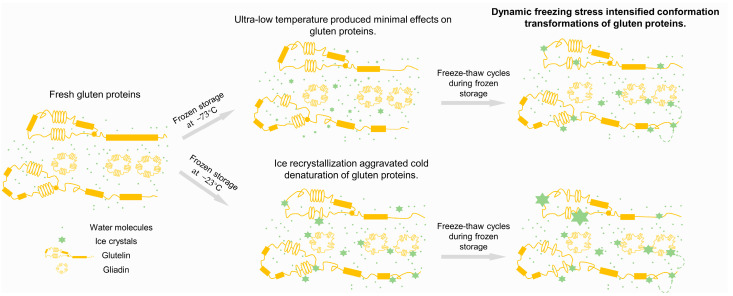
Mechanism of freeze–thaw cycles aggravating the cold denaturation of gluten proteins during frozen storage.

These findings support the mechanistic model proposed in Figure 9, which illustrates how freeze–thaw cycles exacerbate cold denaturation during frozen storage. When gluten proteins are frozen at −23 °C, ice nucleation and growth induce substantial conformational disruptions. In contrast, freezing at −73 °C has minimal effects on protein structure. During continuous storage, proteins maintained at −73 °C experience less cold denaturation due to suppressed ice recrystallization, whereas proteins stored at −23 °C are more susceptible to conformational changes, likely due to increased hydrogen bonding between protein and water molecules. Freeze–thaw cycles simulate temperature fluctuations that repeatedly break and reform hydrogen bonds, thereby amplifying the damaging effects of ice crystals on protein structures. While rapid freezing at −73 °C may partially mitigate these effects, it does not entirely prevent the structural degradation of gluten proteins. These results highlight the importance of both the freezing temperature and temperature stability in minimizing cold-induced denaturation and preserving protein functionality during frozen storage.

## 5. Conclusions

Freeze–thaw cycles were found to accelerate conformational transitions and exacerbate the cold denaturation of gluten proteins during frozen storage. While prolonged freezing reduced the hydration capacity of gluten proteins, the superimposition of freeze–thaw events further intensified this deterioration, as evidenced by decreased water-holding capacity, increased oil-holding capacity, and elevated freezable water content. These changes were accompanied by greater exposure of hydrophobic residues and a marked increase in surface hydrophobicity. Under continuous frozen storage at −73 °C, conformational changes were relatively limited, suggesting effective cryoprotection; however, the introduction of freeze–thaw cycles significantly disrupted structural stability. The α-helix structures were progressively converted into β-sheets and β-turns, driven by a shift toward a more hydrophobic microenvironment. Notably, gluten proteins stored at −23 °C exhibited more pronounced conformational alterations than those stored at −73 °C, and these changes were further amplified by temperature cycling. Molecular dynamics simulations further supported these findings, revealing that hydrogen bond formation and disruption played a central role in mediating cold denaturation. Temperature fluctuations, particularly cycles between 250 K and 300 K, triggered the repeated breaking and reforming of hydrogen bonds between protein–protein and protein–water interfaces, ultimately compromising molecular conformations. Collectively, this study provides mechanistic insights into how freeze–thaw cycles accelerate the cold denaturation of gluten proteins during frozen storage. By linking hydration property with conformational transformations, our findings highlight the critical role of temperature instability in protein destabilization and offer theoretical guidance for designing more effective cryoprotection strategies in frozen food systems.

## Figures and Tables

**Table 1:** Binding parameters for the treated gluten with acrylamide.

Samples	K_sv_ (×10^4^ L/mol)	R^2^	K_b_ (×10^5^ L/mol)	n	R^2^
Control	3.054 ± 0.17 ^a^	0.99	2.57 ± 0.02 ^ab^	1.171 ± 0.02 ^a^	0.98
FS/-73	3.160 ± 0.16 ^ab^	0.99	2.49 ± 0.09 ^a^	1.161 ± 0.04 ^a^	0.99
FS/-23	3.251 ± 0.10 ^abc^	0.99	2.69 ± 0.09 ^c^	1.167 ± 0.03 ^a^	0.99
FST/-73	3.397 ± 0.11 ^bc^	0.99	2.70 ± 0.03 ^c^	1.172 ± 0.03 ^a^	0.99
FST/-23	3.486 ± 0.08 ^c^	0.99	2.67 ± 0.03 ^bc^	1.160 ± 0.03 ^a^	0.99

Means with different letters within the same column are significantly different (*p* < 0.05).

## Data Availability

The original contributions presented in the study are included in the article. Further inquiries can be directed to the corresponding author.

## Abbreviations

The following abbreviations are used in this manuscript:

WHC	Water-holding capacity
OHC	Oil-holding capacity
Δ*H*	Melting enthalpy
MD	Molecular dynamics
RMSD	Root-mean-square deviation
Rg	Radius of gyration
SASA	Solvent-accessible surface area

## References

[1] Arsiccio A., McCarty J., Pisano R., Shea J.-E. (2020). Heightened cold-denaturation of proteins at the ice-water interface. J. Am. Chem. Soc..

[2] Dinani S.T., Carrillo M.F.C., Boom R., van der Goot A.J. (2023). Quality improvement of plant-based meat alternatives by addition of iota carrageenan to pea protein-wheat gluten blend. Eur. Food Res. Technol..

[3] Sun Y., Dong M., Bai J., Liu X., Yang X., Duan X. (2024). Preparation and properties of high-soluble wheat gluten protein-based meat analogues. J. Sci. Food Agric..

[4] Webb D., Li Y., Alavi S. (2023). Chemical and physicochemical features of common plant proteins and their extrudates for use in plant-based meat. Trends Food Sci. Technol..

[5] Jiang L., Zhang H., Zhang J., Liu S., Tian Y., Cheng T., Guo Z., Wang Z. (2024). Improve the fiber structure and texture properties of plant-based meat analogues by adjusting the ratio of soy protein isolate (SPI) to wheat gluten (WG). Food Chem. X.

[6] Xiang N., Yuen J.S., Stout A.J., Rubio N.R., Chen Y., Kaplan D.L. (2022). 3D porous scaffolds from wheat glutenin for cultured meat applications. Biomaterials.

[7] Wang P., Zou M., Li D., Zhou Y., Jiang D., Yang R., Gu Z. (2020). Conformational rearrangement and polymerization behavior of frozen-stored gluten during thermal treatment. Food Hydrocoll..

[8] Liang K., Zhang L., Zeng J., Gao H., Ma H. (2024). Effects of different freezing temperatures on the molecular structure of gluten proteins. J. Food Meas. Charact..

[9] Dai Y., Gao H., Tian X., Huang K., Liu Y., Zeng J., Wang M., Qin Y. (2022). Effect of freeze-thaw cycles at different temperatures on the properties of gluten proteins in unfermented dough. Cereal Chem..

[10] Jia G., Chen Y., Sun A., Orlien V. (2022). Control of ice crystal nucleation and growth during the food freezing process. Compr. Rev. Food Sci. Food Saf..

[11] Li Y., Kong H., Li C., Ban X., Gu Z., Lu Y., Li Z. (2025). Short-clustered maltodextrin mediates stabilization of gluten proteins during frozen storage compared to trehalose and guar gum. Food Chem..

[12] Park J.K., Patel M., Piao Z., Park S.-J., Jeong B. (2021). Size and shape control of ice crystals by amphiphilic block copolymers and their implication in the cryoprotection of mesenchymal stem cells. ACS Appl. Mater. Interfaces.

[13] Lu L., Zhu K.-X. (2023). Physicochemical and fermentation properties of pre-fermented frozen dough: Comparative study of frozen storage and freeze-thaw cycles. Food Hydrocoll..

[14] Tang W., Ye L., Han T., He J., Liu J. (2025). Effect of chitosan with different molecular weights on the freeze-thaw stability of gluten protein: Protein structures, functional characteristics, and cryo-protective mechanism. Food Hydrocoll..

[15] Tang W., Lin X., Ye L., He J., Wang Z., Tang J., Liu J., Zhao P. (2025). Effect of pectin with different esterification degree on the freeze-thaw stability of gluten protein: Structures, functional properties, and cryoprotective mechanism. Food Chem..

[16] Li Y., Li C., Ban X., Cheng L., Hong Y., Gu Z., Li Z. (2021). Alleviative effect of short-clustered maltodextrin on the quality deterioration of frozen dough: Compared with trehalose and guar gum. Food Hydrocoll..

[17] Li Y., Li C., Ban X., Cheng L., Hong Y., Gu Z., Li Z. (2021). New insights into the alleviating role of starch derivatives on dough quality deterioration caused by freeze. Food Chem..

[18] Han C., Ma M., Li M., Sun Q. (2020). Further interpretation of the underlying causes of the strengthening effect of alkali on gluten and noodle quality: Studies on gluten, gliadin, and glutenin. Food Hydrocoll..

[19] Wang P., Xu L., Nikoo M., Ocen D., Wu F., Yang N., Jin Z., Xu X. (2014). Effect of frozen storage on the conformational, thermal and microscopic properties of gluten: Comparative studies on gluten-, glutenin- and gliadin-rich fractions. Food Hydrocoll..

[20] Khrustalev V.V., Poboinev V.V., Stojarov A.N., Khrustaleva T.A. (2019). Microenvironment of tryptophan residues in proteins of four structural classes: Applications for fluorescence and circular dichroism spectroscopy. Eur. Biophys. J..

[21] Li Y., Kong H., Li C., Ban X., Gu Z., Lu Y., Li Z. (2024). Mitigating the effects of starch derivatives on cold denaturation of gluten protein: Insights from hydration capacity and conformation behavior. J. Agric. Food Chem..

[22] Bailey M., Hallett J. (2004). Growth rates and habits of ice crystals between −20 °C and −70 °C. J. Atmos. Sci..

[23] Petzold G., Aguilera J.M. (2009). Ice morphology: Fundamentals and technological applications in foods. Food Biophys..

[24] Wei Q., Zhang G., Xie J. (2024). Alleviative effects of carboxymethyl chitosan on the quality deterioration of frozen rice dough during freeze thaw cycles. Food Hydrocoll..

[25] Zhang H., Fan H., Xu X., Xu D. (2024). Deterioration mechanisms and quality improvement methods in frozen dough: An updated review. Trends Food Sci. Technol..

[26] Wieser H., Koehler P., Scherf K.A. (2022). Chemistry of wheat gluten proteins: Qualitative composition. Cereal Chem..

[27] Davies P.L. (2014). Ice-binding proteins: A remarkable diversity of structures for stopping and starting ice growth. Trends Biochem. Sci..

[28] Ma Y., Hong T., Chen Y., Wu F., Xu X., Jin Z. (2022). The conformational rearrangement and microscopic properties of wheat gluten following superheated steam treatment. Food Control.

[29] Liu M., Chen G., Zhang H., Yu Q., Mei X., Kan J. (2021). Heat-induced inulin-gluten gel: Insights into the influences of inulin molecular weight on the rheological and structural properties of gluten gel to molecular and physicochemical characteristics. Food Hydrocoll..

[30] Liu H., Liang Y., Zhang S., Liu M., He B., Wu X., Yin H., Zhang X., Wang J. (2024). Physicochemical properties and conformational structures of pre-cooked wheat gluten during freeze-thaw cycles affected by curdlan. Food Hydrocoll..

[31] Nawrocka A., Szymańska-Chargot M., Miś A., Wilczewska A.Z., Markiewicz K.H. (2017). Effect of dietary fibre polysaccharides on structure and thermal properties of gluten proteins—A study on gluten dough with application of FT-Raman spectroscopy, TGA and DSC. Food Hydrocoll..

[32] Zhang J., Fan M., Wang L., Qian H., Li Y. (2024). Unveiling the structural and physico-chemical properties of glutenin macropolymer under frozen storage: Studies on experiments and molecular dynamics simulation. Food Res. Int..

[33] Mátyus L., Szöllősi J., Jenei A. (2006). Steady-state fluorescence quenching applications for studying protein structure and dynamics. J. Photochem. Photobiol. B Biol..

[34] Kim S.B., Palmer J.C., Debenedetti P.G. (2016). Computational investigation of cold denaturation in the Trp-cage miniprotein. Proc. Natl. Acad. Sci. USA.

[35] Raghunathan S. (2024). Solvent accessible surface area-assessed molecular basis of osmolyte-induced protein stability. RSC Adv..

[36] Bitonti A., Puglisi R., Meli M., Martin S.R., Colombo G., Temussi P.A., Pastore A. (2022). Recipes for inducing cold denaturation in an otherwise stable protein. J. Am. Chem. Soc..

